# Plastic-binding peptides as anchors for protein scaffolds on synthetic plastics: opportunities and challenges

**DOI:** 10.1039/d5ra06185g

**Published:** 2025-10-27

**Authors:** Sreeahila Retnadhas, Eric L. Hegg, Daniel C. Ducat

**Affiliations:** a Department of Biochemistry and Molecular Biology, Michigan State University East Lansing MI 48824 USA ducatdan@msu.edu; b MSU-DOE Plant Research Laboratory, Michigan State University East Lansing MI 48824 USA

## Abstract

Non-biodegradable synthetic plastics are accumulating in the environment and current recycling methods are limited by harsh processing conditions and inferior quality of recycled products. While biological approaches to plastic degradation offer promise, available enzymes remain inefficient at degrading synthetic plastics due to their inherent physical and chemical properties. Inspired by nature's strategies for degrading resistant natural polymers like cellulose and chitin with enzyme complexes co-localized to polymer surfaces, we quantitatively evaluated a range of previously reported plastic-binding peptides (PBPs) for their capacity to anchor soluble proteins specifically to polystyrene and polypropylene. Among the PBPs tested, LCIM3 demonstrated better specificity when compared to other peptides, binding to polystyrene and polypropylene with an apparent dissociation constant (*K*_D_) in the nanomolar range. By analogy to natural cellulosome complexes, we further investigated the rational design of scaffolds utilizing paired dockerin–cohesin protein binding domains and LCIM3 to enable recruitment of multiple user-defined proteins to plastics. Our study demonstrates that plastic binding peptides were marginally able to increase the localization of proteins and scaffolds onto plastic substrates. However, we also identify a significant non-specific adsorption of many untargeted proteins on plastic as a potential bottleneck for approaches to improve plastic degradation *via* rational engineering. This proof-of-concept study highlights both the opportunities and challenges in designing bio-inspired scaffolds to improve plastic degradation, laying a foundation for future advancements in sustainable plastic recycling and upcycling.

## Abstract 2

Plastic-binding peptides integrated into programmable multienzyme scaffolds validate binding capacity but reveal off-target binding challenges for rational scaffold design.

## Graphical abstract

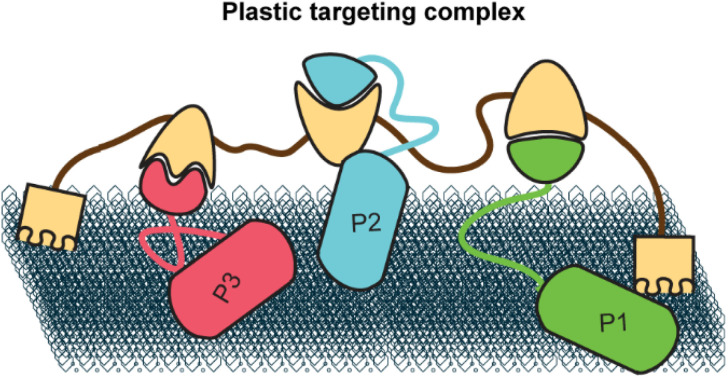

## 1 Introduction

Non-biodegradable synthetic plastics are increasingly replacing traditional materials like metal, glass, and wood due to their chemical inertness, hydrophobicity, lightweight nature, and durability. This widespread shift is evidenced by a steady rise in global plastic production,^1^ and environmental accumulation caused by inadequate waste management practices and plastic's resistance to decomposition. By 2060, this accumulation is projected to triple, surpassing one billion metric tonnes.^2^ The predominant plastics in use today, polyethylene (PE), polypropylene (PP), polyvinyl chloride, polyethylene terephthalate (PET), polyurethane, and polystyrene (PS), are non-biodegradable,^3^ and bioplastics still lack the necessary robustness for widespread application.

Biological recycling and upcycling strategies for plastic waste are of growing interest for their environmental sustainability.^4–6^ However, enzymes capable of degrading synthetic plastics face considerable challenges, especially when acting on recalcitrant polymers dominated by carbon–carbon backbones, such as PE, PS and PP.^6–9^ Potential routes to enhance biological processing of PE, PS and PP can be drawn from recent successes in improving the activity of enzymes capable of hydrolyzing PET (PETases). One potential approach to enhance enzymatic degradation of PET has been to rationally increase binding affinity of enzymes to plastic surfaces, thereby targeting the enzyme and increasing the effective concentration of its intended substrate.^10–14^ To achieve this, several studies have explored fusing PET-degrading enzymes with binding domains, including carbohydrate-binding modules (CBMs), chitin-binding domains (ChBDs), polyhydroxyalkanoate-binding modules (PBMs), hydrophobins, and short peptides such as α-SP: often resulting in reports of improved PET hydrolysis.^10–14^ Similarly, multienzyme scaffolds functionalized with a plastic-binding domain were recently shown to increase PET hydrolysis rates by 6.5-fold compared to an unlinked enzyme mixture.^15^ Multienzyme scaffolds have also been engineered to be displayed on the surface of *Saccharomyces cerevisiae*^16^ and *E. coli*^17^ to develop whole cell catalysts for hydrolyzing PET into its monomers.

Inspired by such findings and by multi-enzyme complexes found in nature for polymer deconstruction, we proposed a similar approach to address the challenge of degrading non-hydrolysable plastics like PS and PP in our recent Perspective.^18^ Herein, we aimed to develop a multienzyme complex for plastic re/upcycling by incorporating plastic binding peptides (PBP) on a programmable protein scaffold. Towards this goal, we investigated published PBPs (a.k.a., anchor peptides, material-binding, or solid-binding peptides) with reported affinity to PP and PS.^19^ Rübsam *et al.*^20^ identified the PS- and PP-binding properties of LCI (liquid chromatography peptide I from *Bacillus subtilis*) and TA2 (tachystatin A2 from *Tachypleus tridentatus*), and subsequently generated a mutant LCIM3 with improved binding properties to these plastics.^21^ Random peptide display libraries have also been used to identify several PS-binding peptides, including HWGMWSY, PS19-6 (RIIIRRIRR), PS19-6L (RLLLRRLRR), PS19 (RAFIASRRIKRP), and PS23 (AGLRLKKAAIHR).^22,23^ Collectively, these studies provide a diverse pool of plastic-binding peptides (PBPs) that can be screened for fusion with plastic-oxidizing enzymes to potentially enhance their catalytic activity.

Despite growing interest in potential applications for PBPs, mechanisms of the interactions between PBPs and plastic surfaces remain poorly characterized. Several reports have demonstrated improved plastic degradation upon fusion of PBPs to enzymes, as summarized in our Perspective.^18^ However, unlike natural polymer-binding domains such as cellulose- or chitin-binding modules, PBPs lack systematic classification and mechanistic studies of their interactions with plastic surfaces. Moreover, quantitative analysis of PBP binding affinities to non-hydrolysable synthetic plastics are rare,^24^ and such studies mostly emphasize interactions between PET^25,26^ and CBMs.^24^ Comprehensive investigations into sequence–function relationships, structure-binding correlations, and comparative binding analyses across different plastics are largely absent. Finally, proteins exhibit a poorly-understood capacity to intrinsically bind/adsorb to plastic surfaces,^27–29^ which has been largely overlooked in the literature for enzymatic plastic recycling. This gap in knowledge makes the rational engineering of plastic-degrading enzymes using PBPs and the interpretation of binding data extremely challenging.

To address gaps in the field, we screened a small library of previously-reported PBPs and quantitatively analyzed their binding properties to PS and PP. We then used the lead PBP to target soluble protein cargo to the surface of PS and PP, followed by constructing a nature-inspired multiprotein complex carrying three soluble protein cargos, akin to natural cellulosomes, to target to PS and PP. Our results suggest significant hurdles in the use of PBPs for rational engineering strategies for plastic degradation. Specifically, while PBPs can enhance PS and PP binding, we find a significant un-targeted protein adsorption from control protein cargos lacking PBPs. Our study highlights a gap in the field: while PBPs are widely reported to improve enzymatic plastic degradation, their contribution is often evaluated qualitatively and not critically assessed in the context of intrinsic protein–plastic interactions. Through systematic, quantitative binding studies, we find the added benefit of PBPs can be marginal, and that many proteins possess inherent plastic-binding capabilities that should be accounted for in rational engineering strategies.

## 2 Experimental

### 2.1 Materials and reagents

Most analytical grade chemicals were purchased from Sigma-Aldrich. 0.05 mm thick additive-free PS films (ST31-FM-000150) and 0.125 mm thick additive-containing PS film (ST31-FM-000125) were obtained from Goodfellow, USA (composition of additives not disclosed by the company). PS microbeads (150 μm) were purchased from Phosphorex, USA. Glass coated 96-well polypropylene plates (60180-P334) were purchased from Thermo Fischer Scientific, USA. 5-FAM conjugated peptides for plastic binding assays were synthesized by Biomatik by solid-phase peptide synthesis; peptides were reported as >95% pure, with additional characterization data from the manufacturer provided as an Appendix to the SI. Gene blocks for mNeonGreen (mNG)-peptide fusion were synthesized by Integrated DNA Technologies, USA and all other genes used in the study were synthesized by Twist Bioscience, USA. Restriction enzymes, DNA polymerase, and other enzymes and reagents for cloning were purchased from New England Biolabs and Thermo Fischer Scientific, USA. Strep-Tactin®XT 4Flow® resin (2-5010-025) for strep affinity protein purification was purchased from IBA lifesciences. HisPur™ Ni-NTA Resin (88222) from Thermo Fischer was used for Ni^2+^ affinity purification of recombinant proteins. The Pierce™ BCA Protein Assay Kit (23225) was used for estimation of protein concentrations.

### 2.2 Cloning, expression and purification of protein fusions for plastic binding assays

mNeonGreen^30^ fusion constructs with peptides listed in Table 1 were individually cloned in pBbA2a with a flexible GGGSGGGS linker between the peptide and mNG and expressed in *E. coli* BL21 (DE3) ArcticExpress for affinity purification (Ni^2+^-NTA). Cell pellets were resuspended in 50 mM Tris (pH 8.0), 100 mM NaCl, protease inhibitor cocktail (Halt™ Protease Inhibitor Cocktail) and 20 mM imidazole and lysed in a high-pressure homogenizer from Constant Systems Ltd. After centrifugation at 10 000 g for 45 min to remove cell debris, His-tagged proteins were purified using Ni^2+^-NTA chromatography (50 mM Tris, pH 8.0, 100 mM NaCl and 250 mM imidazole for elution). Eluted proteins were buffer exchanged into 50 mM Tris (pH 8.0), 100 mM NaCl before estimating protein concentration by Pierce™ BCA Protein Assay Kit. Unfortunately, we observed proteolytic loss of many PBPs during expression, leaving only the mNG fusion tag behind (Fig. S1). Although we changed the expression conditions, which allowed the successful purification of full-length PS23 and LCIM3 fusions, we had limited success with further optimization of purification conditions for the full-length fusion proteins for TA2-M1, AB2, cecropin, and PS19-6.

**Table 1:** List of plastic binding peptides used in the study

Peptides	Uniprot ID	Length	Source	Features
Cecropin (ref. 41)	P01507	37 amino acids	*Hyalophora cecropia*	Unstructured in aqueous, potential to form amphiphilic α-helix
Adenoregulin B2 (ref. 41)	P31107	33 amino acids	*Phyllomedusa bicolor*	Unstructured in aqueous, potential to form amphiphilic α-helix
Tachystatin A2-M2 (ref. 21)	Q9U8X3	44 amino acids	*Tachypleus tridentatus*	Triple stranded amphiphilic β-sheet stabilized by three disulfide bonds
LCIM3 (ref. 21)	P82243	47 amino acids	*Bacillus subtilis*	Four stranded amphiphilic β-sheet with no disulfide bonds
PS19-6 (ref. 23)		9 amino acids	Randomized peptide display library	Three tandem repeats of PS19-6 was used
PS23 (ref. 22)		12 amino acids	Randomized peptide display library	Three tandem repeats of PS23 was used

### 2.3 Design and production of protein fusions for the development of plastic targeting complex

Molecular design of all fusion proteins used for the development of a plastic targeting scaffold and cargo complex is summarized in Fig. S2. Drawing inspiration from cellulosomes, evolutionarily-optimized complexes for cellulose degradation, we designed a backbone scaffold containing three cohesin domains: cohesin 1 – type I first cohesin from CipC (the primary scaffoldin of *Clostridium cellulolyticum*),^31^ cohesin 2 – type I third cohesin from CipA (the primary scaffoldin of *Clostridium thermocellum*), and cohesin 3 – type II first cohesin from OlpB (the anchoring scaffoldin of *C. thermocellum*).^32^ Cohesin subunits were linked sequentially to form the core scaffolding construct using their respective natural C-terminal linkers (Fig. S2). In turn, LCIM3 was fused to both ends of the cohesin scaffold to target it to plastic surfaces; with the N-terminal LCIM3 appended to cohesin 1 using the linker sequence naturally encoded between the CBM and first cohesin of CipC in *C. cellulolyticum*. LCIM3 domains were omitted for control experiments measuring inherent plastic binding of the scaffold.

Three corresponding reporter “cargo” binding partners of the cohesins were designed to evaluate scaffold localization. Specifically, the type I dockerin of Cel5A (endoglucanase from *C. cellulolyticum*), type I dockerin of Cel48S (exoglucanase from *C. thermocellum*), and type II dockerin of CipA (primary scaffoldin from *C. thermocellum*), were fused to three different fluorophores: mNG, mTurquoise2 (mTQ2; PDB ID 3ZTF), and mScarlet (mSL; PDB ID 5LK4), respectively using their natural linkers.

For affinity purification, the plastic-targeting scaffold and the control scaffold were cloned into the pET28a vector with an N-terminal Strep-Tag II. Similarly, dockerin-fusion proteins with fluorescent tags were cloned into pET28a with a C-terminal His-tag for Ni^2+^-based affinity purification. All constructs required for the complex were expressed in *E. coli* BL21 (DE3) cells. Protein expression was induced with 0.1 mM IPTG and carried out overnight at 18 °C to enhance solubility and yield. Following cell lysis, all proteins were purified by passage of the soluble supernatant fraction over the appropriate affinity resin (Strep-Tactin®XT 4Flow® resin for scaffold constructs and HisPur™ Ni-NTA Resin for dockerin cargo reporters) and elution of bound proteins (50 mM biotin for Strep-tagged proteins and 250 mM imidazole elution for His-tagged proteins). An additional gel filtration step was required to purify full-length mTQ2-Cel48S from its degradation products (see Fig. 5B for SDS PAGE of purified protein fusions). After purification, all proteins were buffer exchanged into 25 mM Tris (pH 8.0), 150 mM NaCl, and 5 mM CaCl_2_, and then stored at 4 °C.

### 2.4 Plastic binding assays

#### 2.4.1 Screening of peptides

For initial screening of PBPs, peptides in Table 1 were chemically synthesized conjugated to the fluorophore, 5-FAM (Biomatik). Peptide stocks (10 μM) were prepared by dissolving them in a buffer (50 mM Tris; pH 8.0, 100 mM NaCl), designed to stabilize the peptides (Fig. S3).

Three different forms of PS surfaces were used as substrates to perform initial binding assays – PS film discs, PS microbeads and PS 96-well plates.

##### 2.4.1.1 PS film

Glass-coated 96-well plates were used to perform binding assays involving polystyrene (PS) films. PS film discs, each 3 mm in diameter, were precisely excised from PS sheets using a punching tool. One disc was placed into each well and 5-FAM-conjugated peptides were added to individual wells such that the final assay volume (200 μL) contained 50 mM Tris (pH 8.0), 100 mM NaCl, 1 mg per mL BSA, and 2 μM peptide. The plate was incubated at 30 °C with continuous shaking at 200 rpm to ensure uniform mixing. After a 2-hour incubation, the supernatant was carefully removed using a micropipette, taking care not to disturb PS discs. To eliminate unbound and non-specifically bound peptides, the PS films were washed three times. For each wash, 200 μL of buffer (50 mM Tris, pH 8.0, 100 mM NaCl) was added to each well, the plate was agitated at 30 °C and 200 rpm for 5 minutes, and the wash buffer was then removed. After the final wash, 200 μL of buffer was added to each well, and the residual fluorescence (Ex. 485 nm/Em. 525 nm) was measured.

##### 2.4.1.2 PS beads

Glass-coated 96-well plates were used to perform the PS bead binding assay. Similar to the PS film binding assay, each 200 μL reaction mixture contained 2 μM peptide dissolved in 50 mM Tris (pH 8.0), 100 mM NaCl, and 1 mg per mL BSA. PS microbeads (150 μm), supplied as a 10% suspension in water, were added to a final concentration of 1% (v/v). The plate containing the assay mixture was incubated at 30 °C with shaking at 200 rpm for 2 hours. Following incubation, the beads were washed three times as described above to remove unbound peptides. At each washing step, liquids were carefully removed using a thin-tip micropipette to minimize bead loss. After the final wash, 200 μL of buffer was added to each well, and the residual fluorescence (Ex. 485 nm/Em. 525 nm) was measured as described previously.

##### 2.4.1.3 PS plate

96-Well plates made of PS (Griener Bio-One, 655101) and PP were directly used as another form of plastic substrate for binding assays. The wells were first washed with 50 mM Tris (pH 8.0), 100 mM NaCl before adding 2 μM of 5-FAM conjugated peptides (50 mM Tris (pH 8.0), 100 mM NaCl, 1 mg per ml BSA) followed by incubation at 30 °C for 2 h and washing three times with buffer as discussed above. After washing, the empty plates were excited at 485 nm, and the emission was measured at 525 nm using a plate reader.

#### 2.4.2 Estimation of apparent dissociation constant (*K*_D_)

Plastic binding assays to estimate the apparent dissociation constants (*K*_D_) were performed by titrating synthesized 5-FAM conjugated peptides (or fusion constructs or complexes) over a concentration range of 0 to 2 μM in 96-well plates made of polystyrene (PS) and polypropylene (PP), following the method described above. The data was fitted into either one-site specific binding model (eqn (1)) or one-site total binding model (eqn (2)) using GraphPad Prism to estimate *K*_D_ of peptides against the target plastics PP and PS1
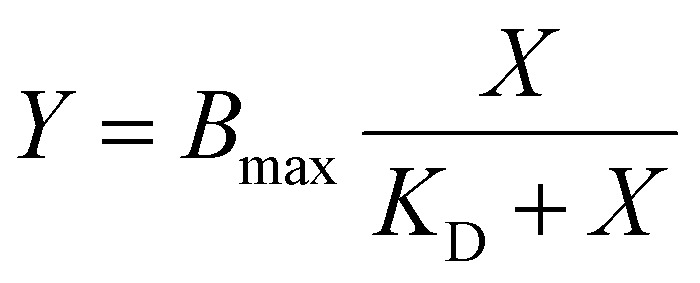
2
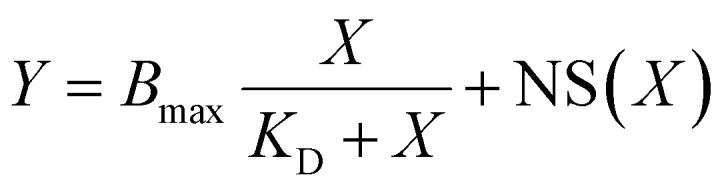
where, *B*_max_ – maximum specific binding, *K*_D_ – equilibrium dissociation constant, NS – slope of nonspecific binding, *X* – peptide concentration and *Y* – residual fluorescence measured.

### 2.5 Protein binding to PS or PP plates

Assessment of the binding of PBP-tagged reporter proteins to PS or PP was conducted directly on 96-well plates manufactured of the appropriate polymer material. The procedure followed was similar to that described in Section 2.4.1.3 for assessing peptide binding to PS plates. Increasing concentrations of proteins/complexes (prepared in 50 mM Tris, pH 8.0, 100 mM NaCl, and 1 mg per mL BSA) up to 1 μM were added to the appropriate plate (PS or PP) and incubated at 30 °C for 2 h. The wells were washed three times as described in Section 2.4.1.3, and residual protein levels were quantified using a plate reader with the following excitation/emission wavelengths: mNG – 500/540 nm, mTQ2 – 430/475 nm, mSL – 570/610 nm. Analysis was typically conducted both for a LCIM3-fused protein paired with a control protein lacking the LCIM3 domain to assess for plastic binding that could not be attributed to the fused PBP.

### 2.6 Scaffold complex assembly, purification, and plastic binding assay

Assembly of the cohesin-based scaffold with the three dockerin cargo constructs was conducted by mixing one part of scaffold (or control scaffold) with 1.2 parts of mNG-Cel5A dockerin, mTQ2-Cel48S dockerin, and mSL-CipA dockerin. Proteins were combined in the above molar ratio in Tris/NaCl buffer supplemented with calcium chloride (25 mM Tris pH 8.0, 150 mM NaCl, and 5 mM CaCl_2_). The mixture was incubated at 4 °C overnight to facilitate complex formation. The following day, the assembled protein complex was allowed to bind to Strep-Tactin®XT 4Flow® resin by incubating the protein mixture with the resin for 30 minutes at 4 °C under mild rocking conditions. After binding, the resin was washed thoroughly with the same Tris buffer (25 mM Tris pH 8.0, 150 mM NaCl) to remove unbound proteins and free dockerin constructs. The bound complex was then eluted using three column volumes of 50 mM biotin prepared in a buffer containing 50 mM Tris pH 8.0, 5 mM CalCl_2_ and 100 mM NaCl. The eluted complex was immediately buffer-exchanged into 25 mM Tris, 150 mM NaCl, and 5 mM CaCl_2_ for storage.

The interaction between the scaffold and the three fluorophore–dockerin constructs was assessed using a magnetic pull-down assay with MagStrep® Strep-Tactin®XT beads (IBA Lifesciences). Following the manufacturer's instructions, 100 μL of bead suspension was equilibrated with 50 mM Tris buffer (pH 8.0, 100 mM NaCl) and subsequently resuspended in 250 μL of protein mixture in the ratio mentioned above. The mixture was incubated at 4 °C for 30 minutes on a rocker to promote binding to the beads. As the scaffold was the only construct containing a Strep-tag, it was specifically captured by the beads. After incubation, unbound proteins were removed by placing the tube in a magnetic separator and discarding the supernatant. The beads were then washed with Tris buffer (50 mM Tris, 100 mM NaCl, pH 8.0) to eliminate residual unbound proteins before eluting the bound scaffold with 150 μL of 50 mM biotin prepared in 50 mM Tris buffer.

Assembled scaffold complexes were assessed for binding to PS and PP as described in 2.5.

## 3 Results

### 3.1 Comparative analysis of multiple plastic binding peptides

**Fig. 1:**
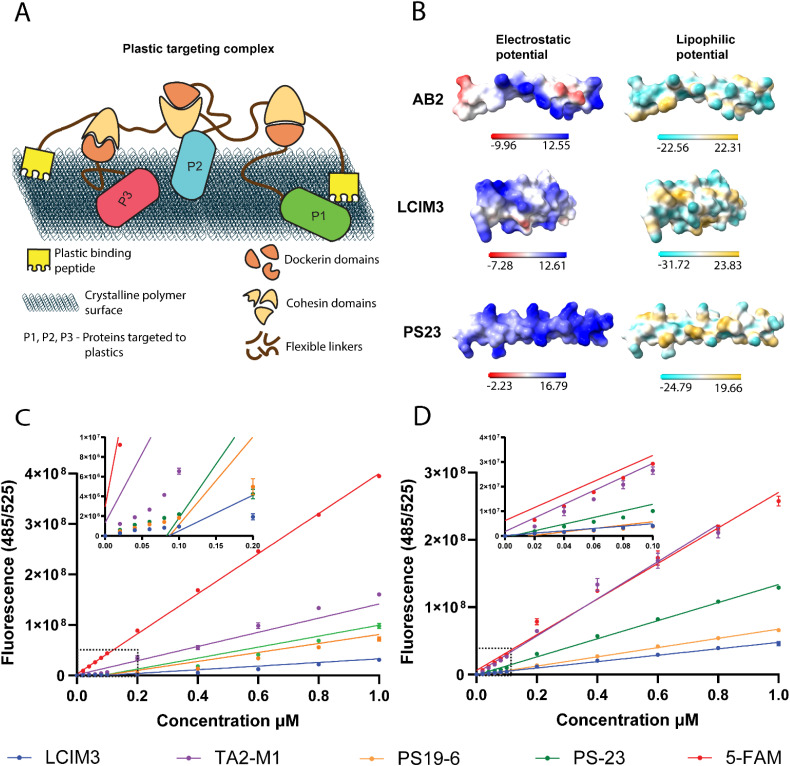
(A) Cartoon schematic of an engineered scaffold which can be localized to plastic surfaces using plastic binding peptides (PBPs; yellow) and possesses cohesin domains to recruit target proteins with corresponding dockerin domains. (B) Surface charges and hydrophobicity of representative plastic binding peptides from diverse groups (α-helical or β-sheet, natural peptides or synthetic peptides). Most plastic binding peptides in the literature have acidic and amphiphilic surfaces. (C) Fluorescent standard of chemically synthesized PBPs conjugated to 5-FAM in the absence of BSA. Fit of standard curve is poor, especially due to deviating trends at low concentrations (inset) of PBP–dye conjugates. (D) Fluorescent standard of PBP–dye conjugates in the presence of 1 mg per mL BSA as a crowding agent.

Towards development of a plastic-targeting multienzyme complex, we first screened selected PBPs from the literature (Table 1), focusing on peptides previously described to bind to one or both of the recalcitrant synthetic polymers, PP and PS. Cecropin A and AB2 are unstructured peptides in aqueous solutions but are hypothesized to form amphiphilic α-helical structures when they come in contact with lipid membranes or membrane-mimicking environment.^33,34^ Both TA2 and LCI form stable amphiphilic β-sheet structures: TA2 with three disulfide bonds^35^ and LCI without any disulfide bonds.^36^ PS19-6 and PS23 are both synthetic peptides with high affinity to PS that were identified from randomized peptide display screens.^22,23^ Despite their structural diversity, all of the listed peptides share two key features: a net positive charge under physiologically relevant conditions and amphiphilic character (Fig. 1B and S4). These shared features are proposed to contribute to peptide adsorption onto plastic surfaces,^37^ as supported by mutational studies showing improved binding upon substitution of acidic residues with basic, polar, or non-polar amino acids.^21,38^ Additionally, *in silico* predictive simulations suggest that plastic binding may be enhanced by amino acids with bulky side chains and alternating hydrophobic and hydrophilic patches.^39,40^ However, exact mechanisms by which the positive charge of PBPs enhances binding to the highly crystalline, hydrophobic surfaces of PS and PP is not yet established.

Previous studies identifying these peptides or employing them to enhance plastic-targeting properties have primarily relied on qualitative analyses, with limited attention given to their binding kinetics. To address this gap, we conducted a quantitative analysis of their binding behavior as a foundation for targeting soluble proteins and complexes to PS and PP surfaces. We began by screening previously reported variants of LCI and TA2 (LCIM3 and TA2-M1), which were engineered for improved plastic-binding properties,^21^ along with the originally-reported sequences of other peptides listed in Table 1.

For this purpose, a subset of four peptides (LCIM3, TA2-M1, PS19-6, and PS23) were chemically synthesized and conjugated to the fluorophore 5-carboxyfluorescein (5-FAM). A simple titration of the free fluorophore yielded a reliable standard curve across the tested concentration range (20 nM to 1000 nM), showing a strong linear relationship at all points (Fig. 1C). However, when the 5-FAM-conjugated peptides were titrated, fluorescence readings became inconsistent at low peptide concentrations (≤0.05 μM), with significant deviation from the linear trend observed at higher concentrations (Fig. 1C). We suspected molecular crowding effects might contribute to this behavior, as such effects are known to influence protein folding and stability. We therefore included a classic crowding agent, bovine serum albumin (BSA), to determine if this agent would correct for unexpected behavior of synthesized peptide–dye conjugates. Inclusion of 1 mg per mL BSA in all dilution buffers (Fig. 1D and S5B) effectively mitigated the issue, restoring consistent fluorescence trends even at low concentrations. Due to similar artifacts observed with PBP–protein fusions at low concentrations (Fig. S5A), we included 1 mg per mL BSA as a crowding agent in all subsequent experiments to minimize the impact of protein instability on determination of binding affinity. It should be noted that our results suggest that molecular crowding is an important factor to consider when evaluating PBP properties, although this has been often omitted in other studies evaluating plastic binding protein domains.

### 3.2 Estimation of apparent dissociation constant (*K*_D_) for PBP–fluorophore conjugates

**Fig. 2:**
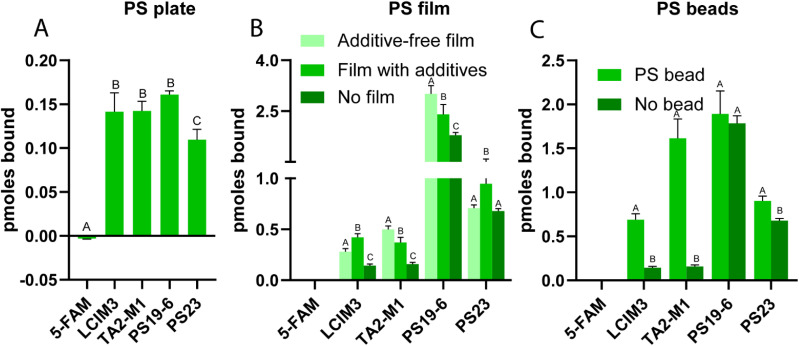
Conjugation of a PBP domain to fluorescence dye alters attachment to polymer surfaces. Binding of 2 μM of indicated PBP–dye conjugates to (A) PS well plate, (B) PS film, and (C) PS microbeads. The 96-well plate made of PS yielded more reproducible binding data compared to the PS film and PS microbeads. PS19-6 and PS23 exhibited non-specific interactions with the glass-coated wells of the 96-well plates used for the assay with PS film and PS beads. Data points labelled with different letters are significantly different (*P* < 0.05) by Tukey's multiple comparison test. Bars in B and C with the same letter within the same peptide do not differ statistically (*P*-value > 0.05) by Tukey's multiple comparison test.

We sought to directly compare selected PBPs and to evaluate their relative binding affinities to a range of substrates while developing a methodology that could control for potential non-specific peptide interactions with polymers. We examined peptide binding to three different forms of PS materials (beads, films, and plates) to determine the most suitable substrate for plastic-binding assays. Initial evaluation was performed in PS 96-well plates *via* incubation of 2 μM peptide and subsequent evaluation of residual fluorescence associated with plastic materials following a series of wash steps to remove unbound peptide. A fluorescent dye-only reference was used as a negative control. In agreement with prior studies, we found that all PBP-5-FAM conjugates were retained in part on the PS plate, while the dye alone did not exhibit measurable PS binding (Fig. 2A). We next utilized other PS substrates to validate these results, including PS beads milled to a uniform size (150 μm) and PS film discs (3 mm in diameter). LCIM3 and TA2-M1 conjugates still displayed binding to PS beads and discs (relative to paired controls; Fig. 2B and C). However, PS19-6 and PS23 exhibited significant levels of non-specific binding to other surfaces, like the glass-coated well plates used for the assay (Fig. 2B and C). When controlling for the background level of non-specific attachment to the glass plate, LCIM3 and TA2-M1 exhibited the most reliable PS binding properties across all tested peptides (Fig. S6). Furthermore, PBP binding assays using PS plates were relatively reproducible, while PS film and PS beads exhibited increased variability. Factors contributing to experimental noise likely included non-specific attachment of PBPs to glass-coated wells and technical considerations, such as bead loss during wash steps, or the buoyancy of PS beads/film which could interfere with substrate submersion during incubation. We therefore conducted most subsequent binding experiments on 96-well plates made of PS and PP.

**Fig. 3:**
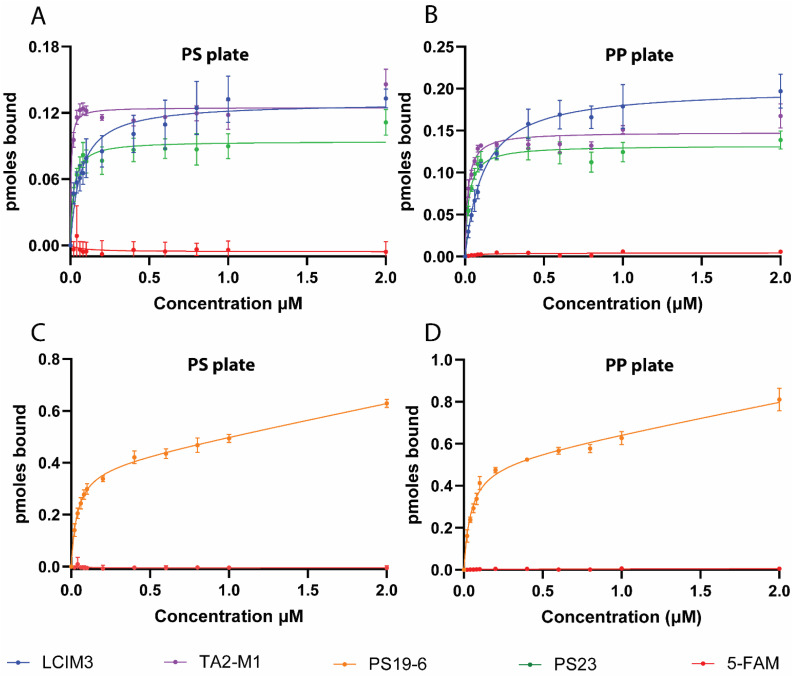
Binding isotherms of PBP–dye conjugates to PS and PP. Binding of LCIM3, TA2-M1 and PS23 to (A) polystyrene (PS) and (B) polypropylene (PP) fitted to a one-site specific binding model. Binding of PS19-6 to (C) polystyrene and (D) polypropylene fitted to one-site total binding model. The one-site specific binding model assumes monolayer adsorption on solid surfaces, whereas the one-site total binding model accounts for both specific adsorption to the surface and non-specific binding to previously bound ligands, resulting in potential multilayer attachment.

We next utilized the optimized methodology to estimate *K*_D_ values of synthetic peptides by titrating them against PP and PS well plates. Binding data for the peptides LCIM3, TA2-M1, and PS23 to PS and PP well-plates were fit with a one-site specific binding model (eqn (1) equivalent to Langmuir's adsorption isotherm) (Fig. 3A and B), indicating that the data supports a binding modality of peptides forming a monolayer on plastic surfaces with constant adsorption energy. Furthermore, the total number of peptides bound to PS or PP at saturating concentrations was similar for LCIM3, TA2-M1, and PS23. By contrast, the binding data of PS19-6 to both PP and PS plates fit poorly to a Langmuir assumption instead exhibiting higher fit with a one-site total binding model (eqn (2) similar to Freundlich's model) (Fig. 3C and D). The one-site total binding model assumes that ligands may non-specifically attach to both an absorptive surface and to other bound ligands, forming a multi-layer attachment to the surface. This is reflected in the continued linear increase in binding of PS19 beyond the saturation concentration, the slope of which is represented by NS in eqn (2) (Fig. 3C and D). According to the *K*_D_ estimated by fitting binding data to either Langmuir's model or Freundlich's model, TA2-M1 has the highest affinity to PS and PP, followed by PS23, PS19-6, and LCIM3 (Table 2). Notably, all tested peptides demonstrated apparent *K*_D_ values in the nanomolar-range.

**Table 2:** Apparent dissociation constants (*K*_D_) of chemically synthesized peptides and mNG-LCIM3^a^

	*K*_D_	
	PS (nM)	PP (nM)
LCIM3	64 ± 10	173 ± 8
TA2-M1	4 ± 1	17 ± 1.6
PS19-6	37 ± 3.2	42 ± 4
PS23	20 ± 3	22 ± 2
mNG-LCIM3	260 ± 63	176 ± 34

a Binding data from three independent experiments, each performed duplicate, were analyzed by fitting the data either to a one-site specific binding model (LCIM3, TA2-M1, PS23, and mNG-LCIM3) or to a one-site total binding model (PS19-6). *K*_D_ data represented as mean ± standard error.

### 3.3 PBP adaptor domains enhance recruitment of soluble proteins to plastic surfaces

To measure the capacity of PBPs to recruit larger protein constructs to plastic polymers, we constructed expression cassettes of all six peptides from Table 1 with the fluorescent reporter protein, mNeonGreen (mNG). Unfortunately, while most mNG–PBP fusions could be heterologously expressed, only LCIM3-fusion proteins remained as a full-length proteins while other peptide constructs exhibited considerable protein degradation and/or insolubility under a range of different expression conditions (Fig. S1; see Section 2.2). We therefore proceeded with only LCIM3-functionalized proteins for subsequent experiments.

**Fig. 4:**
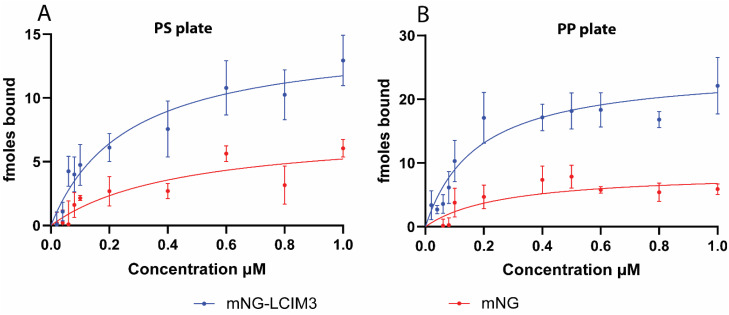
Recruitment of soluble proteins *via* PBP dosmains. Binding isotherms of mNG encoded with or without a LCIM3 to (A) polystyrene and (B) polypropylene well-plates. mNG-LCIM3 binds to both PS and PP surfaces and shows a better fit to the adsorption isotherm model, whereas the control construct, mNG alone, exhibits a poor fit with larger standard errors (see Table 3).

We evaluated the capacity of LCIM3 to increase plastic binding capacity of conjugated soluble proteins by titrating mNG-LCIM3 and mNG against PS and PP well-plates. We observed that appending the LCIM3 increased calculated affinity and saturation concentration of mNG-LCIM3 to both PS and PP relative to mNG alone (Fig. 4A and B). Binding data showed sufficient fit to a one-site specific binding model. The *K*_D_ of mNG-LCIM3 to PP was estimated to be 176 ± 34 nM, which is similar to observations for the chemically synthesized LCIM3 conjugated to 5-FAM (*K*_D_: 173 ± 8 nM, Table 2). Binding affinity of mNG-LCIM3 to PS was lower for the mNG fusion construct (*K*_D_: 260 ± 63 nM, Table 2) in comparison to the chemically synthesized peptide (*K*_D_: 64 ± 10 nM), but still significantly stronger than the mNG control (Table 3). The mNG control also exhibited poor curve fitting (Fig. 4), as indicated by a high standard error (Table 3), suggesting it does not follow a typical adsorption isotherm. Similar trends were observed using PS microbeads as the binding substrate (Fig. S7), indicating LCIM3 can enhance targeting of soluble proteins to different forms of plastics. However, it is noteworthy that the total amount of mNG-LCIM3 bound to PP and PS substrates was in femtomoles, as opposed to the picomoles observed with chemically synthesized LCIM3 incubated on an identical surface area of plastic. It is possible that the larger size of the mNG-LCIM3 fusion protein (∼35 kDa) may contribute to the lower carrying capacity relative to the smaller chemical dye.

**Table 3:** Apparent dissociation constants (*K*_D_) of fusion proteins used in the study^a^

Protein	Fluorescence measured Ex/Em (nm)	*K*_D_	
		PS (nM)	PP (nM)
mNG	485/525	444 ± 346	266 ± 103
mNG-LCIM3	485/525	260 ± 63	176 ± 35
Complex	500/540	42 ± 6	45 ± 5
	570/610	40 ± 13	198 ± 44
Control complex	500/540	190 ± 20	134 ± 9
	570/610	118 ± 67.5	816 ± 285
mNG-Cel5A dockerin	500/540	351 ± 52	217 ± 29
mSL-CipA dockerin	570/610	265 ± 49	313 ± 42

a Binding data from three independent experiments, each performed in duplicate, were analyzed by fitting to a one-site specific binding model. *K*_D_ data represented as mean ± standard error.

### 3.4 Targeting a multi-subunit complex to plastic surfaces

#### 3.4.1 Design of synthetic multi-subunit plastic targeting complex

**Fig. 5:**
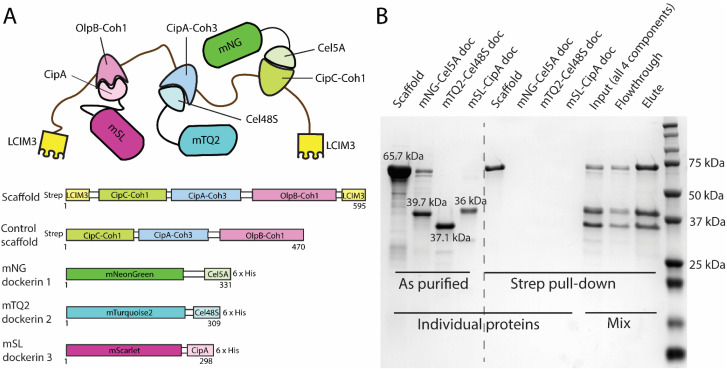
Purification and assembly of a synthetic cohesin–dockerin scaffold complex. (A) The molecular design of all fusion proteins used for the development of the plastic-targeting complex. A plastic-targeting complex was designed using three cohesins from cellulosomal proteins (CipC, CipA, OlpB) and fused to LCIM3 on both termini. A control scaffold lacking LCIM3 was also constructed. Dockerins from Cel5A, Cel48S, and CipA were fused to distinct fluorophores *via* native linkers. Scaffold constructs carried an N-terminal Strep II tag; dockerin fusions had a C-terminal 6×His tag. (B) Assembly of the dockerin–cohesin complex was validated by pull-down experiments targeting the scaffold backbone using MagStrep® Strep-Tactin® XT beads. Lanes to the left of the dotted line show the purified protein fusions used for complex assembly. Lanes to the right of the dotted line represent pull-down results. The first four lanes on the right demonstrate that only the scaffold binds to the Strep-Tactin XT beads when each protein is incubated individually. When all four proteins are incubated together, the fluorophore–dockerin fusions co-elute with the scaffold (elute lane) in the Strep-Tactin XT pull-down.

LCIM3 demonstrates potential for targeting soluble proteins to the surfaces of PS and PP, which led us to explore the use of LCIM3 to direct a multi-protein complex to PS and PP surfaces. To construct the complex, we employed three specific cohesin–dockerin pairs, as detailed in Section 2.6 (summarized in Fig. 5A). Briefly, the scaffold was designed by fusing three cohesin domains derived from native cellulosome targeting-scaffolds (*i.e.*, the first cohesin from CipC, third cohesin from CipA, and first cohesin from OlpB). To target the scaffold to PS/PP substrates, LCIM3 domains were appended on the N- and C-termini (Fig. 5A and S2). A control scaffold was similarly constructed with the three cohesins and linkers, but without the LCIM3 tags. Reporter “cargo” proteins were designed by fusing fluorescent proteins mNG, mTQ2, and mSL to three compatible dockerin domains (to form mNG-Cel5A dockerin, mTQ2-Cel48S dockerin, and mSL-CipA dockerin) which specifically bind their cognate cohesins on the scaffold and the control scaffold. These dockerin-fluorescent tag protein fusions carried a C-terminal 6×His tag for purification *via* Ni^2+^-NTA affinity chromatography (see Section 2.6, Fig. 5A and S2). Complexes of the scaffold (or control scaffold) and the three dockerin–fluorophore fusion proteins were assembled as described in Section 2.5. We next confirmed that the dockerin–cohesin pairs were sufficient to enable binding of the cargo to the synthetic scaffold. We first incubated purified scaffold with an excess of dockerin cargo (see Section 2.7), to allow binding of scaffold cohesin domains to the corresponding dockerin–cargo proteins. To verify appropriate assembly of the scaffold–cargo complex, we conducted pulldown experiments targeting the Strep II-affinity tag found solely on the synthetic scaffold using Strep-Tactin magnetic beads (Fig. 5B). As expected, recovered scaffold co-precipitated with dockerin fusion cargo proteins in an approximately equi-molar ratio (mNG-Cel5A dockerin, mTQ2-Cel48S dockerin, and mSL-CipA; elute lane, Fig. 5B). The eluted complex exhibited fluorescence at three distinct excitation/emission wavelengths, 500/540, 430/475, and 570/610, corresponding to the fluorophores mNG, mTQ2, and mSL, respectively, confirming the presence of all three dockerin-containing fluorophores bound to the scaffold (Fig. S8). By contrast, dockerin–cargo did not react with Strep-Tactin magnetic beads in the absence of the synthetic scaffold, verifying the cohesin–dockerin pairs were sufficient to recruit the intended cargo to the scaffold (Fig. 5B). Size-exclusion chromatography of the assembled and purified complex revealed three distinct peaks (Fig. S9): one at ∼450 kDa (likely representing an oligomerized/aggregated complex), one at ∼200 kDa (corresponding to the expected molecular weight of the full complex), and one at ∼160 kDa (consistent with incomplete complexes lacking one of the three dockerins). These results demonstrate that the assembly of the scaffold with three fluorescent proteins is successful and can be directly utilized for plastic-targeting experiments.

#### 3.4.2 Binding of assembled complex to PS and PP plates

**Fig. 6:**
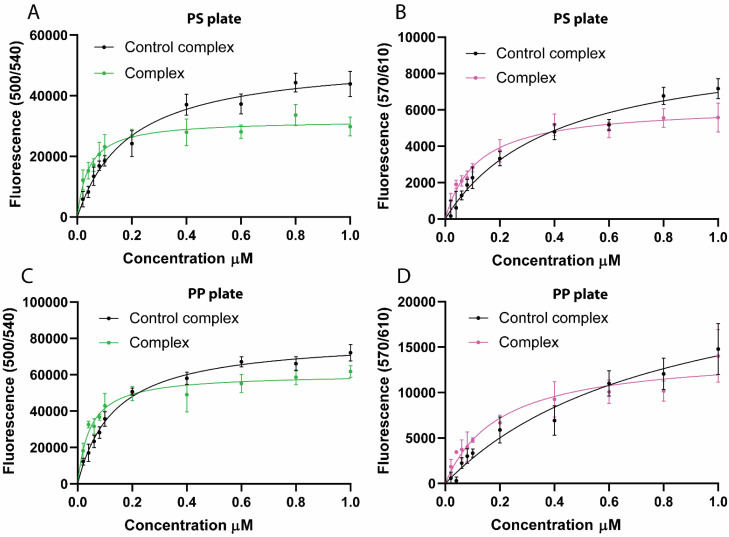
Binding of synthetic scaffold to PS and PP. Binding of the plastic-targeting complex (with LCIM3) and the control complex (without LCIM3) to plastic surfaces was assessed by measuring fluorescence from the same assay plate at two different excitation/emission wavelength pairs, corresponding to the two distinct fluorescent reporters incorporated into the complex. (A) PS plates at 500/540 nm (excitation/emission; mNG-dockerin), (B) PS plates at 570/610 nm (mSL-dockerin), (C) PP plates at 500/540 nm, and (D) PP plates at 570/610 nm.

Purified scaffold–dockerin complexes were used to evaluate their binding to PS and PP surfaces. Following the binding assay, fluorescence was measured at three excitation/emission wavelength pairs to assess the recruitment of dockerin-tagged fluorescent cargo. Binding data from the mNG-dockerin reporter (500/540 nm) was analyzed using a one-site specific binding model to estimate the apparent *K*_D_ (Table 3, Fig. 6A and C). Based on this data, LCIM3 was able to increase the affinity of the complex by 4.5-fold for PS and 2.9-fold for PP compared to the control complex. Data from mSL-dockerin reporter (670/610 nm) showed similar binding trends (Table 3, Fig. 6B and D), while sensitivity limitations in our plate-reader instrumentation at 430/475 nm precluded reliable analysis of the binding of the mTQ2-Cel48S dockerin cargo. Taken together, our data suggests that a cohesin scaffold can successfully bind different dockerin–cargo proteins, and that LCIM3 PBPs can marginally increase the apparent affinity of the complex to PS and PP.

### 3.5 Non-specific interactions of proteins with PS and PP

**Fig. 7:**
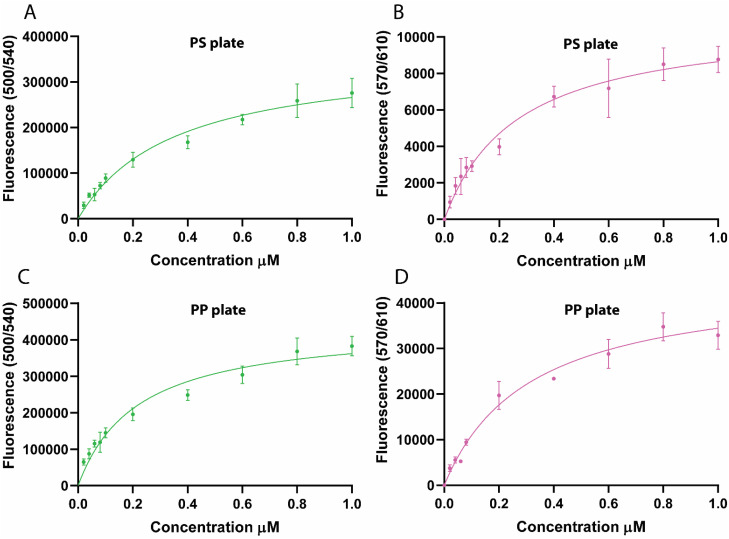
Non-specific plastic adsorption of fluorophore–dockerin reporters. Non-specifc binding of (A) mNG-Cel5A dockerin to PS plate, (B) mSL-CipA dockerin to PS, (C) mNG-Cel5A dockerin to PP plate, (D) mSL-CipA dockerin to PP.

While our data showed that LCIM3 could consistently increase the affinity of both soluble proteins and multi-subunit complexes to the surface of PS and PP, some inconsistencies in the binding data of the assembled scaffold–cargo complex prompted further investigation. For instance, the apparent *K*_D_ of the assembled scaffold complex was distinct depending on if it was estimated using the fluorescence properties associated with either the mNG-Cel5A or mSL-CipA dockerin cargo (Table 3). Futhermore, although the control scaffold complex does not contain any plastic-binding peptide (PBP), the binding data fit well to the adsorption model with affinities to PS and PP estimated in the nanomolar range (Fig. 6 and Table 3). We therefore evaluated the capacity of just the dockerin cargo proteins to attach to PP and PS in the absence of the targeting scaffold complex. These controls revealed that the dockerin–fluorophore fusions alone could bind to PS and PP surfaces (Fig. 7A–D). The inherent affinity of the dockerin cargo to PS and PP was significantly lower than under conditions where the targeting scaffold was present. However, it is notable that the calculated *K*_D_ of dockerin cargo (mNG-Cel5A or mSL-CipA) were comparable to that of the PBP-tagged protein (*i.e.*, mNG-LCIM3) reported previously (Fig. 4, 7, Tables 2 and 3).

## 4 Discussion

In this work, we evaluated the potential of published plastic-binding domains to develop a strategy for targeting plastic-degrading enzymes to PS and PP, with the long-range goal of determining if these domains may provide a method for rational design of scaffolding analogous to those enzyme complexes targeted to recalcitrant polymers in nature. We evaluated six PBPs described in the literature, which exhibit similar surface properties including amphiphilic surface amino acids and net positive charge under physiologically relevant conditions (Fig. 1B and S3). Our results validate the capacity of PBPs to bind to different plastic substrates, but also highlight significant limitations requiring additional consideration to enable rational engineering strategies to functionally target plastic-degrading enzymes to plastic polymers. We find that multiple PBPs exhibit considerable levels of non-specific binding to off-target materials and may decrease protein the expression/stability of fusion proteins. Notably, the intrinsic capacity of untargeted proteins to adsorb to PS and PP may significantly impact the success of enzyme-targeting strategies to these recalcitrant polymers.

To fill a gap in the literature on PBPs, we began by quantitative analysis of the apparent binding affinities of six lead PBPs previously reported in the literature (Table 1). All tested PBPs enhanced the apparent affinity of a non-reactive dye (5-FAM) to PP and/or PS when compared to a dye-only control, although some peptides exhibited features better suited for protein-targeting applications. After initial screening, LCIM3 was selected as the binding peptide for further studies due to its ease of heterologous expression and specific binding properties compared to other peptides tested. In contrast, PS23 and PS19-6 proved particularly challenging to work with because of their non-specific adsorption to off-target materials (Fig. 2B and C), while other peptides displayed practical limitations related to their expression and proteolytic degradation (Fig. S1). LCI is one of the most extensively studied PBPs to date, and has been utilized in applications including microplastic biosensors,^42,43^ plastic degradation,^21,44^ agriculture,^45,46^ and antifouling agents.^47,48^ Curiously, while the estimated *K*_D_ values for LCIM3 fused to a chemical dye (LCIM3-5FAM; 173 ± 8 nM) and a protein (mNG-LCIM3; 176 ± 34 nM) were nearly identical on PP plates, the same constructs displayed disparate binding values to PS plates (mNG-LCIM3; 260 ± 63 nM compared to LCIM3-5FAM; 64 ± 10 nM). This discrepancy underscores the complexity of protein–plastic interactions, suggesting that features of the protein cargo itself, and not merely the binding properties of a given PBP, influence its attachment to PS and PP. Building on the capacity of LCIM3 to target soluble proteins to plastic surfaces, we explored its application in directing a cellulosome-based scaffold carrying three soluble proteins (mNG, mTQ2, and mSL) to PS and PP surfaces. Appending LCIM3 conferred modest but significant improvement in the affinity of the scaffolding complex for PS and PP (4.5-fold and 2.9-fold, respectively), compared to the control complex (*i.e.*, no LCIM3). However, we consistently found that proteins lacking PBPs bind to plastics with high affinities in the nanomolar range. This included the control scaffold on PS (190 ± 20 nM) and control fluorophore–dockerin constructs (200–400 nM; Table 3). Notably, these apparent affinities are similar to that of other soluble proteins where a PBP was appended (*e.g.*, mNG-LCIM3 fusion; 260 ± 63 nM, Table 3). Taken together our data show that the contribution of PBPs to binding PS and PP may be limited, especially when considered against the significant intrinsic plastic-binding properties of untargeted proteins, which we find commonly display nanomolar-range affinities. Such broad and non-specific absorption of proteins to plastic surfaces presents a barrier to engineering strategies targeting enzymes with plastic-targeting sequences as well as the design of higher-order plastic-degrading enzyme complexes.

The mechanisms underlying protein adherence to synthetic plastic surfaces remain poorly understood – a critical knowledge gap that limits the development of strategies to overcome non-specific interactions. Short-range interfacial forces, such as van der Waals interactions, are one common explanation of observed non-specific binding of proteins to plastics.^49^ Such interactions may be particularly influenced by amino acids with hydrophobic R-groups, as examined in some targeted studies.^21^ Moreover, *in silico* peptide design studies suggest that the most effective plastic-binding peptides are enriched in bulky amino acids and feature a combination of hydrophilic and hydrophobic regions,^39^ characteristics also observed in the peptides used in this study (Fig. S4).

Another proposed model for protein binding to plastic surfaces involves partial or complete unfolding of proteins upon contact with solid materials, exposing their hydrophobic cores. A recent study showed that several unrelated proteins adsorb onto synthetic polymer surfaces such as PET and HDPE.^29^ While the driving forces behind this non-specific adsorption remain unclear, the authors suggested that adsorbed proteins may predominantly exist in an unfolded (and catalytically inactive) state, in equilibrium with their folded form.^29^ Interestingly, various studies have shown that protein unfolding occurs during adsorption onto solid materials, including plastics, with the degree of unfolding depending on both the protein's intrinsic properties and the type of material.^50–54^ There is currently no clear evidence to determine whether the degree of unfolding observed in these binding studies is sufficient to inactivate enzymes. For instance, earlier research on hexokinase demonstrated that adsorbed hexokinase retained its catalytic activity on polypropylene tubes at the air–liquid interface,^55^ while other studies have primarily focused on enzyme adsorption without addressing its effects on catalytic activity or binding reversibility.^56–58^ Similarly, our study herein shows that reporter proteins lacking a PBP targeting domain can adsorb to PS and PP while retaining their fluorescent properties, which would be unexpected if these proteins have been largely unfolded. Furthermore, the reversibility of protein binding on plastic substrates *via* such intrinsic pathways is unknown, a consideration that has substantial implications for the repeated rounds of binding and unbinding that would be required for catalytic efficiency. Uncertainty in the mechanisms behind the non-specific attachment of proteins to plastics underscore the need for additional foundational research to enable rational engineering approaches for biological recycling of synthetic plastics. Specifically regarding the results reported here, either model of non-specific protein binding may partially explain the apparently high affinity of some of our constructs. For example, we observe that the control scaffold complex (lacking LCIM3) exhibits an unexpectedly high-affinity of binding to PS and PP (Table 3) relative to the PBP-targeted construct mNG-LCIM3 (Table 2). One possibility is that the larger molecular size (∼164 kDa) of the scaffold relative to mNG-LCIM3 (∼35 kDa), presents a greater surface area for van der Waals interactions. Alternatively, the relatively unstructured “linker” regions between the globular cohesin domains may present more opportunities to initiate a binding event leading to partial protein unfolding. More broadly, without foundational knowledge of the mechanisms at protein–plastic interfaces, unlocking the full potential of PBPs for plastic processing applications may be hindered.

## 5 Conclusions

Binding domains in natural polymer degradation systems such as cellulases, chitinases, and polyhydroxybutyrate depolymerases provide a blueprint for enhancing the degradation of synthetic polymers. Integrating these domains into depolymerizing enzymes has been shown to improve catalytic efficiency for materials like PET and PLA.^18,44^ Building on this concept, our findings demonstrate that plastic-binding proteins (PBPs) can increase the affinity of soluble proteins and protein scaffolds to polystyrene (PS) and polypropylene (PP) surfaces, with binding affinities comparable to those of PET hydrolases for PET.^10,59^ However, not all PBPs display equal promise for rational approaches because some exhibit significant off-target interactions with other materials, which may lead to enzyme loss during the treatment of mixed plastic waste. Importantly, many unrelated proteins and peptides show intrinsic affinities to PS and PP with apparent *K*_D_ similar to those of PBPs. Rational engineering of plastic targeting enzymes and enzyme complexes will likely require methods to decrease the inherent and unpredictable binding of non-specific proteins to PS and PP.

## Author contributions

The manuscript was written through contributions of all authors. All authors have given approval to the final version of the manuscript. S. R. contributed to the experimental design, data collection, data analysis, and writing of the original draft. E. L. H. and D. C. D. contributed to the conceptualization, experimental design, data analysis, and review and editing of the manuscript.

## Conflicts of interest

There are no conflicts to declare.

## Supplementary Material

RA-015-D5RA06185G-s001

## Data Availability

The data supporting this article have been included as part of the supplementary information (SI), including certificates of analysis for the synthetic peptides commercially purchased from Biomatik in the Appendix. Raw data files used to compile figures (*e.g.* Excel worksheets) are available upon request by contacting the corresponding author. Supplementary information is available. See DOI: https://doi.org/10.1039/d5ra06185g.
