# Supplementary material for: Plastic-binding peptides as anchors for protein scaffolds on synthetic plastics: opportunities and challenges
Source: RSC Adv. 2025 Oct 27;15(48):41115–28. doi: 10.1039/d5ra06185g (PMC12558382; doi:10.1039/d5ra06185g)
Supplement: RA-015-D5RA06185G-s001 [file RA-015-D5RA06185G-s001.pdf]

Supplementary Information (SI) for RSC Advances.
This journal is © The Royal Society of Chemistry 2025

Supporting Information

Plastic-Binding Peptides as Anchors for Protein Scaffolds on Synthetic Plastics: Opportunities and Challenges

Sreeahila Retnadhas[a], Eric L Hegg[a], Daniel C Ducat[*, a, b]

[a] Department of Biochemistry and Molecular Biology, Michigan State University, East Lansing, MI, 48824, United States

[b] MSU-DOE Plant Research Laboratory, Michigan State University, East Lansing, MI, 48824, United States

[*] Corresponding author: ducatdan@msu.edu

Table of Contents:

Supplementary Figures S1-S9

Appendix – Peptide synthesis characterization data

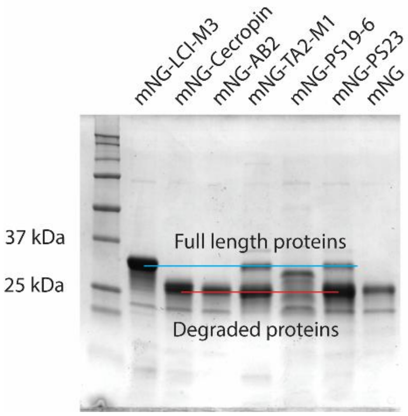

Fig. S1: Purification of mNG-PBP fusions (SDS PAGE). Among the fusion constructs expressed in *E. coli*, mNG-LCI (35 kDa) was the only one to exhibit robust expression of the full-length protein. In contrast, mNG-cecropin (33.5 kDa) and mNG-AB2 (32.7 kDa) underwent significant degradation. Similarly, mNG-TA2-M1 (33.5 kDa), mNG-PS19-6 (33.2 kDa), and PS23 (33.5 kDa) showed a small fraction of full-length protein in the purified samples, with the majority of protein bands migrating at the size of mNG alone (27 kDa).

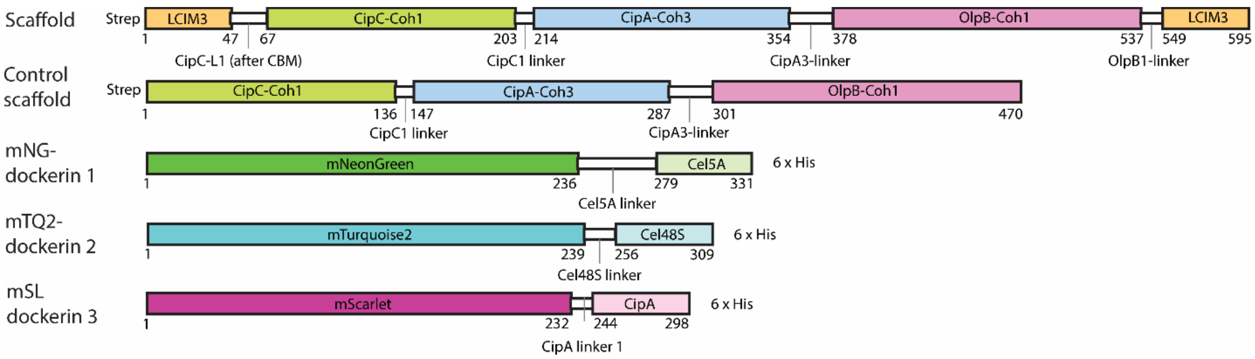

Fig. S2: Molecular design of fusion proteins used for the development of the plastic-targeting complex. A plastic-targeting complex was designed using components of native cellulosomes. The LCIM3 PBP was encoded at both ends of the backbone scaffold in place of a carbohydrate-binding module (CBM). Cohesin-dockerin interactions are known to be species- and type-specific: type I cohesins from *Clostridium thermocellum* specifically bind to type I dockerins from the same organism, while type II cohesins have specific affinity for type II dockerins from the same organism. Accordingly, we used one type I cohesin from *Clostridium cellulolyticum* (first cohesion domain from the protein CipC), one type I cohesin from *C. thermocellum* (third cohesin domain from CipA), and one type II cohesin from *C. thermocellum* (first cohesin domain from OlpB). These cohesins were linked sequentially using their natural C-terminal linkers. LCIM3 and CipC-Coh1 were connected using the N-terminal natural linker of CipC-Coh1. A control scaffold was constructed with all three cohesins but without LCI-M3. The corresponding dockerins for the cohesins were derived from two endoglucanases (Cel5A and Cel48S) of the respective species and the type II dockerin from CipA. All scaffold backbone constructs included a Strep II tag at their N-terminus, while the dockerins were tagged with a 6xHis sequence at their C-terminus, following optimization for expression.

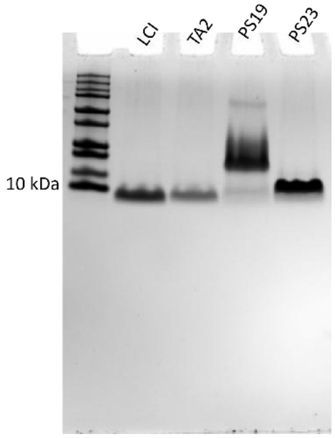

Fig. S3: SDS PAGE of chemically synthesized peptides (synthesized by Biomatik): Lyophilized powder of peptides received from Biomatik was dissolved in tris buffer (50 mM Tris; pH 8.0, 100 mM NaCl) before running on a denaturing SDS PAGE to verify purity.

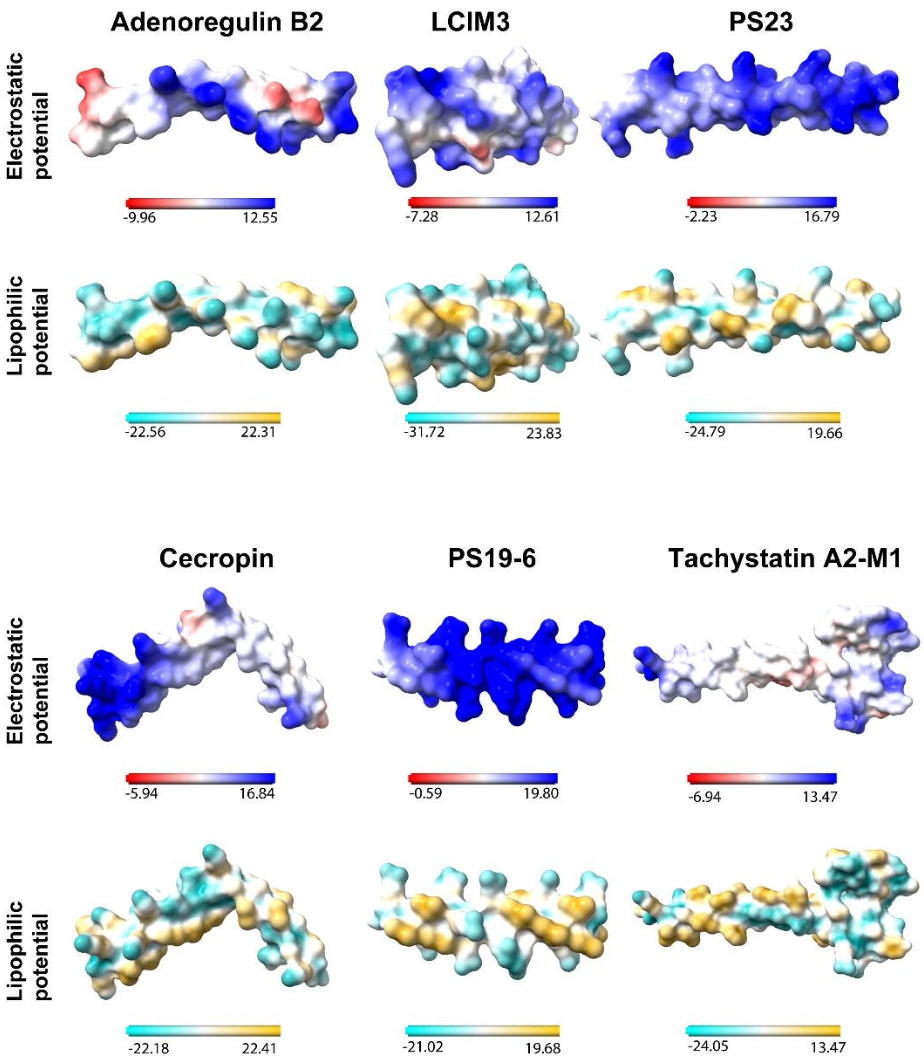

Fig. S4: Predicted surface electrostatic potential and surface lipophilic potential of plastic-binding peptides. AlphaFold structures were used to visualize surface potentials in ChimeraX. These peptides exhibit amphiphilic amino acids on their surfaces, which may facilitate plastic binding through hydrophobic interactions. Additionally, a notable feature is their high cationic surface potential, which could contribute to their binding properties by unknown mechanisms.

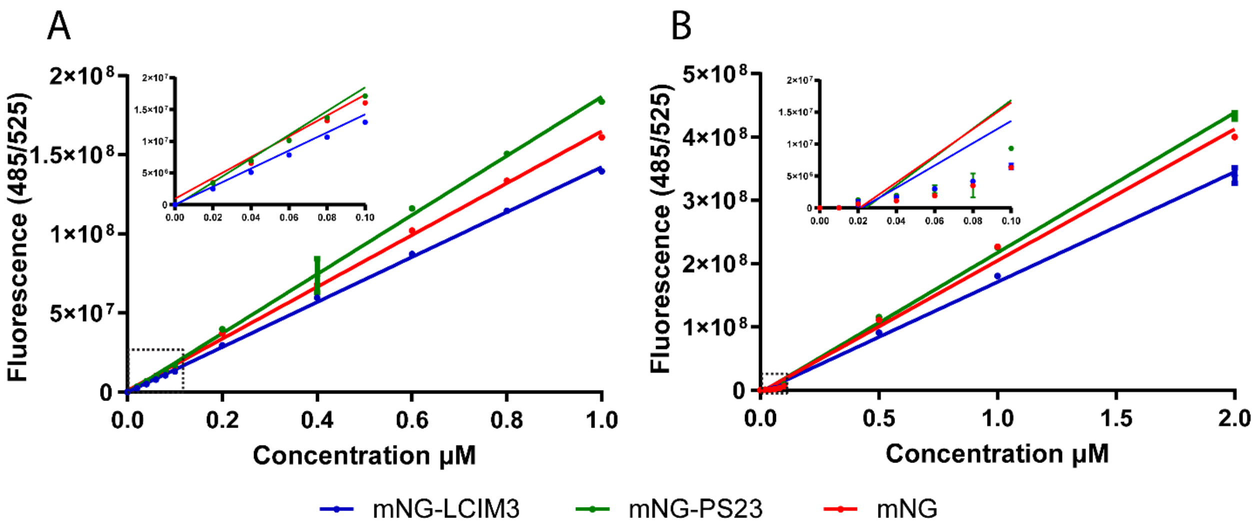

Fig. S5: Molecular crowding artifacts observed in mNG fusion constructs. Fluorescence standards of mNG fusion proteins (mNG-LCIM3, mNG-PS23, and mNG) were measured under (A) in the presence of 1 mg/mL BSA as a crowding agent and (B) in the absence of BSA. Measured fluorescence at low protein concentrations deviated from the linear regression line of the standard curve, as depicted in the inset. Molecular crowding effects may be particularly important to account for in plastic-binding studies, as exposed hydrophobic regions of proteins at low concentrations can contribute to non-specific hydrophobic interactions with plastic surfaces.

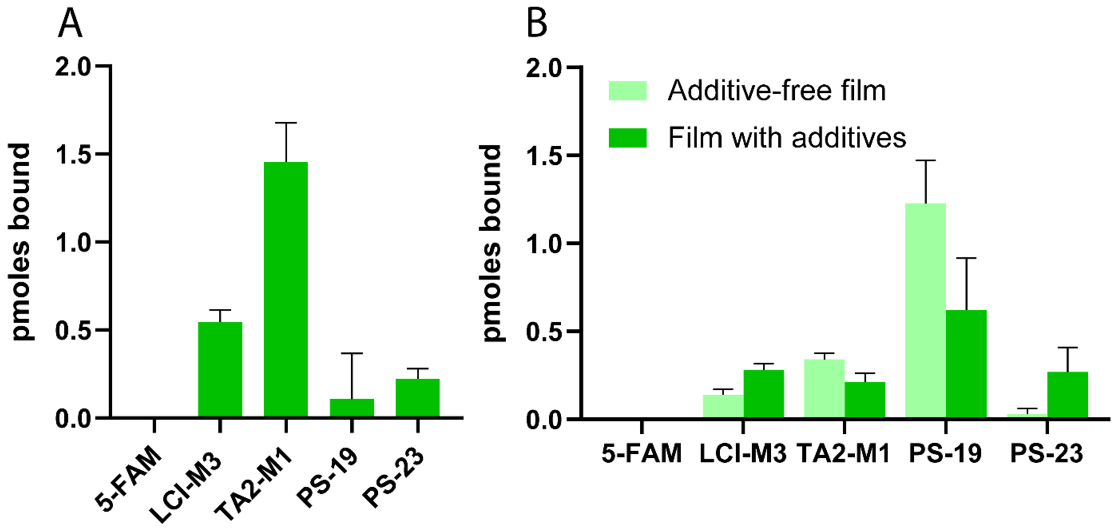

Fig. S6: Calculated binding of PBPs to different plastic substrates. The binding of synthesized peptides to (A) PS microbeads and (B) PS films after accounting for the non-specific binding of peptides to the glass-coated wells of a 96-well plate (*i.e.*, total residual PBP-dye conjugates minus "blank" residual conjugates in control reactions lacking plastic substrates). PS19-6 and PS23 exhibited minimal binding to PS beads, while PS films showed reduced binding to LCI-M3 and TA2-M1.

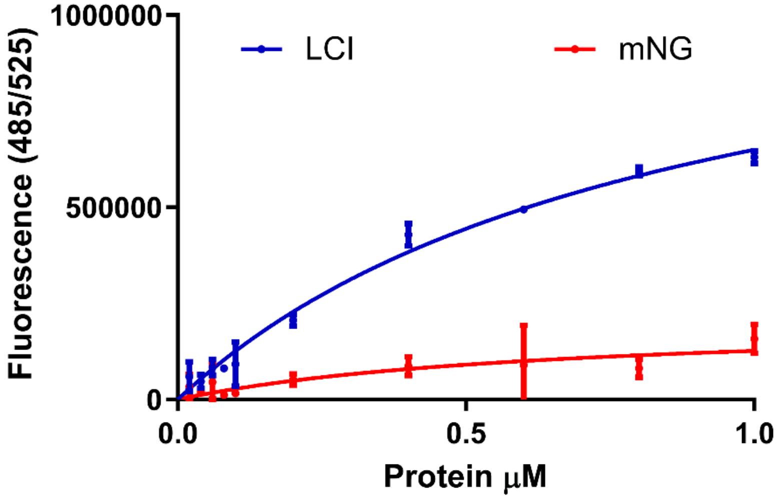

Fig. S7: Binding of mNG-LCI-M3 to PS microbeads. LCI-M3 effectively targets mNG to PS microbeads, exhibiting a binding curve similar to that observed with PS plates. The apparent K_D was estimated to be 131 ± 40.8 nM.

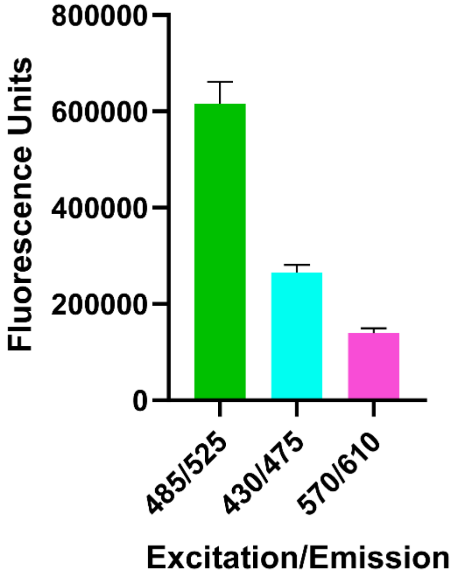

Fig. S8: Fluorescence measurement of 10 nM assembled and purified scaffold complex. The complex was formed by assembling Scaffold, mNG-Cel5A dockerin, mTQ2-Cel48S dockerin, and mSL-CipA dockerin in a molar ratio of 1: 1.2: 1.2: 1.2 overnight at 4°C in the presence of 5 mM $CaCl_2$. Fluorescence at all three excitation/emission wavelengths show the successful incorporation of all three dockerin cargos.

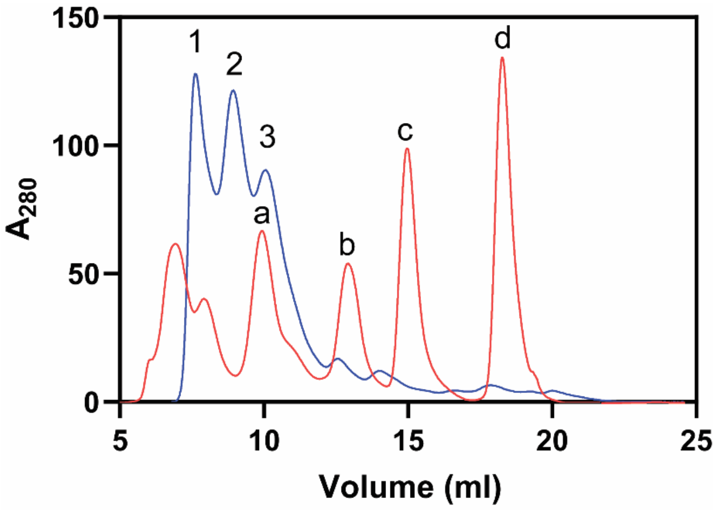

Fig. S9: Size-exclusion chromatography of the fully assembled scaffold complex. Purified scaffold complex was analyzed by size-exclusion chromatography (blue line) revealed three distinct peaks, likely corresponding to oligomerized fraction (peak 1 at ~450 kDa), complete complex (peak 2 at ~200 kDa) and incomplete complexes with any two dockerins bound (peak 3 at ~160 kDa). Size exclusion standard (red line) labeled with peak a, b, c and d corresponds to 158 kDa, 44 kDa, 17 kDa and 1.3 kDa respectively.

Biomatik
Tel: (519) 489-7195, (800) 836-8089
Fax: (519) 231-0140, (877) 221-3515
Email: info@biomatik.com
http://www.biomatik.com

Certificate of Analysis

**Date:**	2023-05-24
**Order Number:**	#SP230487
**Product Type:**	Chemically synthesized peptide
**Catalog Number:**	1070167
**Peptide Name:**	LCIM3
**Sequence (N to C):**	5-FAM-AIKLVQSPNGNFAASFVLDGTKWIFKSKRYDSSKRYWVGIYEVWDRK
**MW:**	5914.59
**Salt Form:**	Trifluoroacetate (TFA Salt)
**Quantity:**	5.2mg
**Suggested Solvent:**	1.0mg peptide soluble in 1.0ml DMSO
**Lot Number:**	P230406-YW1070167
**Appearance:**	Yellow lyophilized powder.
**Storage:**	Store lyophilized peptide at -20°C upon receipt. Reconstitute only the amount of peptide needed for immediate use.
**Limited Usage:**	For Research Use Only. Not for use in diagnostic procedures, or for administration to humans or animals.

**ASSAY**	**SPECIFICATION**	**ACTUAL**
**MW by MS**	5913.60	Conforms
**Purity by HPLC**	>95%	96.41%
**Peptide Content**	N/A	N/A
**TFA Content**	N/A	N/A
**Moisture Content**	N/A	N/A

Biomatik
Tel: (519) 489-7195, (800) 836-8089
Fax: (519) 231-0140, (877) 221-3515
Email: info@biomatik.com
http://www.biomatik.com

MS REPORT

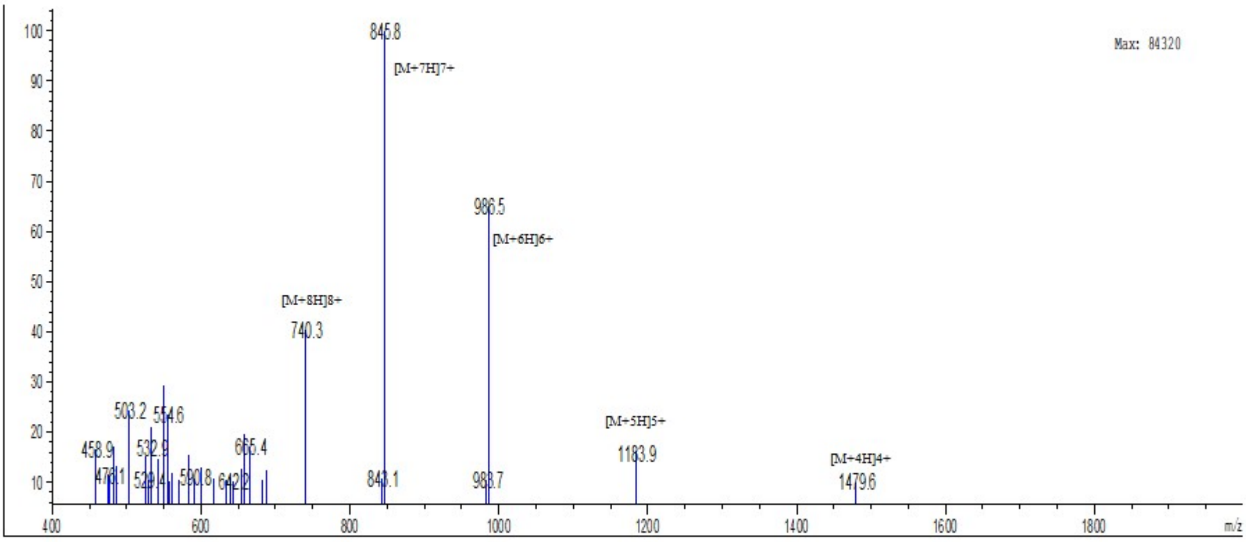

Sample Description		Instrument	Agilent-6125B		
Analyzed date:	2023-05-15	Probe:	ESI	Probe Bias:	+ 4.5kv
Analyst:	YU	Nebulizer Gas Flow:	1.5L/min	Detector:	1.5kv
Sample:	LCIM3 AK-47	CDL:	-20.0v	T. Flow:	0.2ml/min
M.W.:	5914.59	CDL Temp.:	250 °C	B. Conc.:	50%H2O/50%ACN
Lot. No.:	P230406-YW1070167	Block Temp.:	200 °C		

Biomatik
Tel: (519) 489-7195, (800) 836-8089
Fax: (519) 231-0140, (877) 221-3515
Email: info@biomatik.com
http://www.biomatik.com

HPLC REPORT

Structure : LCIM3 AK-47
Number : 010250011
Lot No : P230406-YW1070167
Column : 4.6×250mm,KLM PS/DVB
Solvent A : 0.1% trifluoroacetic in 100% acetonitrile
Solvent B : 0.1% trifluoroacetic in 100% water

Gradient :	A	B
0.01min	20%	80%
25min	60%	40%
25.1min	100%	0%
30min	STOP	

Flow rate : 1.0 mL/min
Wavelength : 220nm
Volume : 5ul

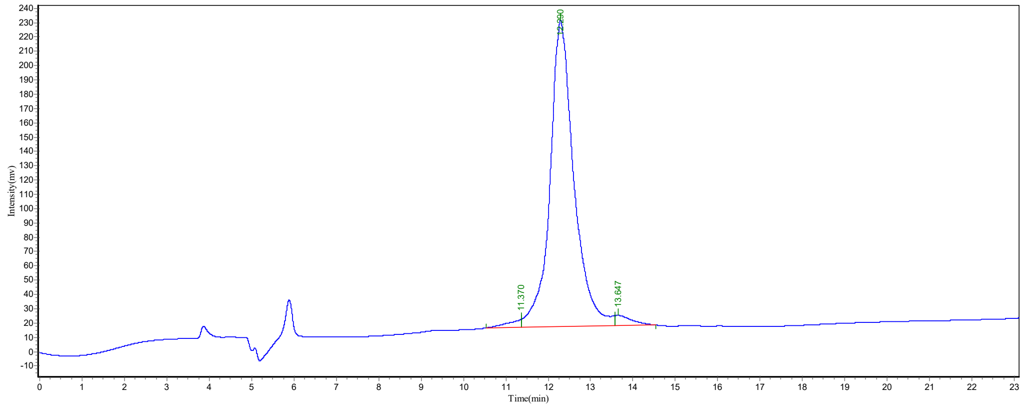

Peak No.	Ret Time	Height	Area	Conc.
1	11.370	5716.004	122041.375	1.3973
2	12.290	214356.141	8420826.000	96.4119
3	13.647	7352.056	191354.063	2.1909
Total				100.000

Biomatik
Tel: (519) 489-7195, (800) 836-8089
Fax: (519) 231-0140, (877) 221-3515
Email: info@biomatik.com
http://www.biomatik.com

Certificate of Analysis

**Date:**	2023-05-24
**Order Number:**	#SP230487
**Product Type:**	Chemically synthesized peptide
**Catalog Number:**	1070170
**Peptide Name:**	PS-19
**Sequence (N to C):**	5-FAM-RIIIRRIRRRIIIRRIRRRIIIRRIRR
**MW:**	4076.99
**Salt Form:**	Trifluoroacetate (TFA Salt)
**Quantity:**	5.2mg
**Suggested Solvent:**	1.0mg peptide soluble in 1.0ml H2O
**Lot Number:**	P230406-YW1070170
**Appearance:**	Yellow lyophilized powder.
**Storage:**	Store lyophilized peptide at -20°C upon receipt. Reconstitute only the amount of peptide needed for immediate use.
**Limited Usage:**	For Research Use Only. Not for use in diagnostic procedures, or for administration to humans or animals.

**ASSAY**	**SPECIFICATION**	**ACTUAL**
**MW by MS**	4076.80	Conforms
**Purity by HPLC**	>95%	96.01%
**Peptide Content**	N/A	N/A
**TFA Content**	N/A	N/A
**Moisture Content**	N/A	N/A

Biomatik
Tel: (519) 489-7195, (800) 836-8089
Fax: (519) 231-0140, (877) 221-3515
Email: info@biomatik.com
http://www.biomatik.com

MS REPORT

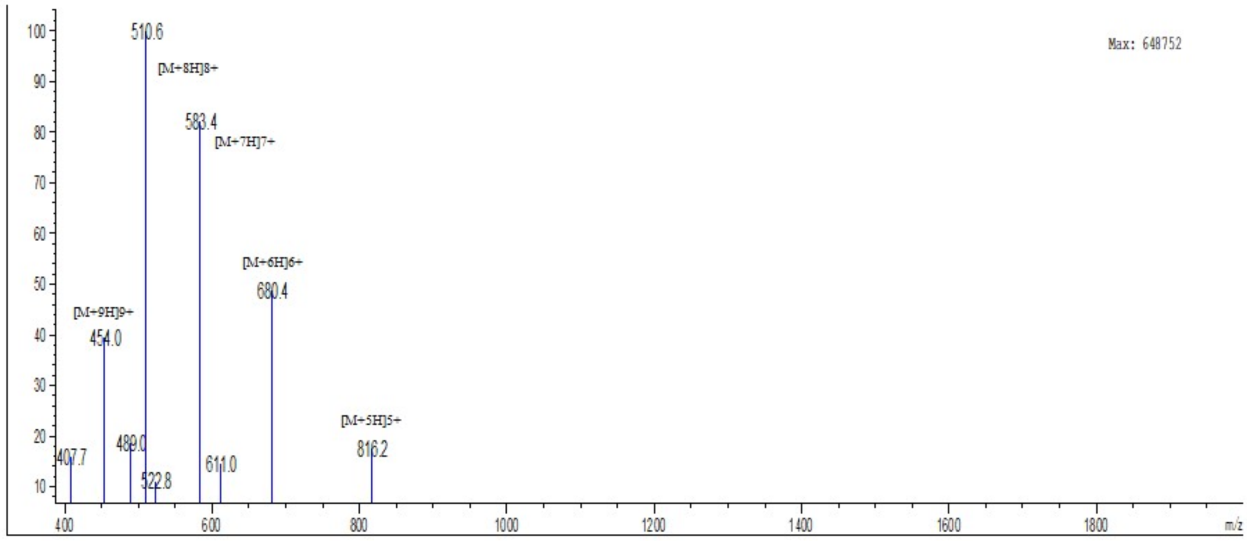

Sample Description		Instrument	Agilent-6125B		
Analyzed date:	2023-05-15	Probe:	ESI	Probe Bias:	+4.5kv
Analyst:	YU	Nebulizer Gas Flow:	1.5L/min	Detector:	1.5kv
Sample:	PS-19 RR-27	CDL:	-20.0v	T. Flow:	0.2ml/min
M.W.:	4076.99	CDL Temp.:	250 °C	B. Conc.:	50%H2O/50%ACN
Lot. No.:	P230406-YW1070170	Block Temp.:	200 °C		

Biomatik
Tel: (519) 489-7195, (800) 836-8089
Fax: (519) 231-0140, (877) 221-3515
Email: info@biomatik.com
http://www.biomatik.com

HPLC REPORT

Sample Description:
Structure : PS-19 RR-27
Number : 0200046
Analyst : HCM
Lot No : P230406-YW1070170
Column : 4.6mm*250mm, SinoChrom ODS-BP
Solvent A : 0.1% trifluoroacetic in 100% acetonitrile
Solvent B : 0.1% trifluoroacetic in 100% water
Gradient :

	A	B
0.01min	25%	75%
25min	50%	50%
25.1min	100%	0%
30.0min	STOP	

Flow rate : 1.0ml/min
Wavelength : 220nm
Volume : 5ul

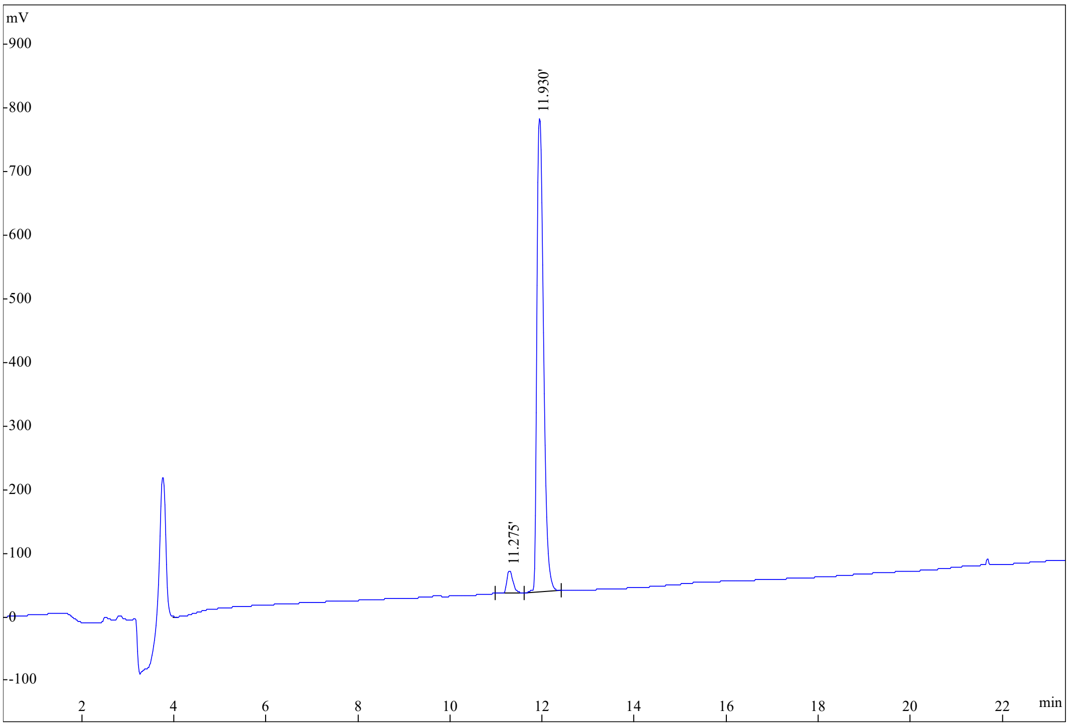

Rank	Time	Conc.	Area	Height
1	11.275	3.987	280777	34812
2	11.930	96.01	6762080	743405
Total		100	7042857	778217

Biomatik
Tel: (519) 489-7195, (800) 836-8089
Fax: (519) 231-0140, (877) 221-3515
Email: info@biomatik.com
http://www.biomatik.com

Certificate of Analysis

**Date:**	2023-05-17
**Order Number:**	#SP230487
**Product Type:**	Chemically synthesized peptide
**Catalog Number:**	1070169
**Peptide Name:**	PS-23
**Sequence (N to C):**	5-FAM-AGLRLKKAAIHRAGLRLKKAAIHRAGLRLKKAAIHR
**MW:**	4323.15
**Salt Form:**	Trifluoroacetate (TFA Salt)
**Quantity:**	5.6mg
**Suggested Solvent:**	1.0mg peptide soluble in 1.0ml (H2O:Acetonitrile=8:2)
**Lot Number:**	P230406-YW1070169
**Appearance:**	Yellow lyophilized powder.
**Storage:**	Store lyophilized peptide at -20°C upon receipt. Reconstitute only the amount of peptide needed for immediate use.
**Limited Usage:**	For Research Use Only. Not for use in diagnostic procedures, or for administration to humans or animals.

**ASSAY**	**SPECIFICATION**	**ACTUAL**
**MW by MS**	4323.20	Conforms
**Purity by HPLC**	>95%	96.53%
**Peptide Content**	N/A	N/A
**TFA Content**	N/A	N/A
**Moisture Content**	N/A	N/A

Biomatik
Tel: (519) 489-7195, (800) 836-8089
Fax: (519) 231-0140, (877) 221-3515
Email: info@biomatik.com
http://www.biomatik.com

MS REPORT

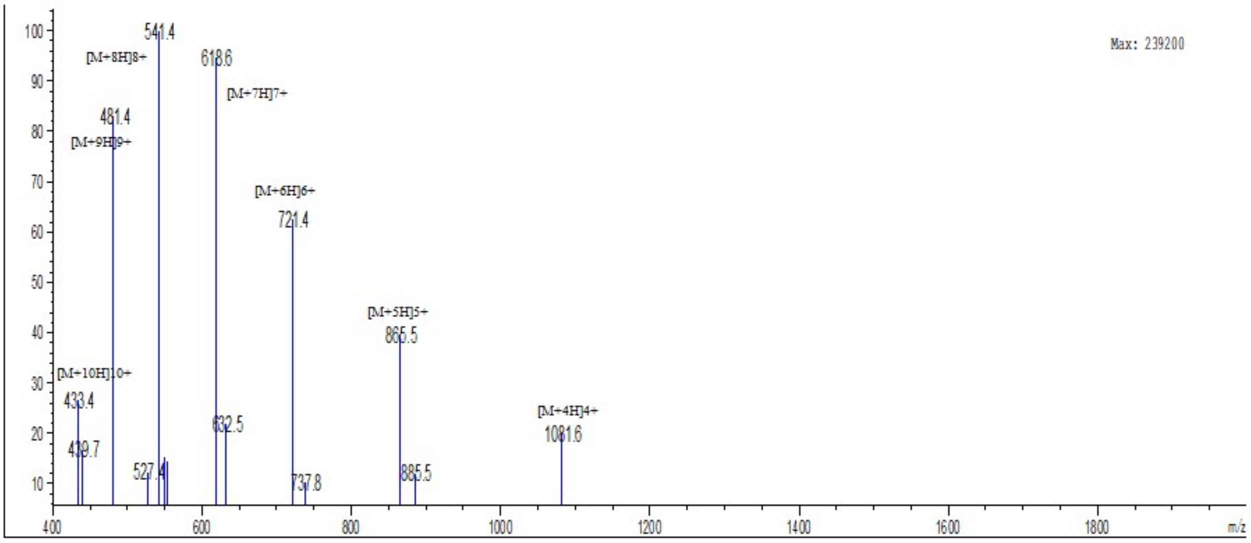

Sample Description		Instrument	Agilent-6125B		
Analyzed date:	2023-05-12	Probe:	ESI	Probe Bias:	＋4.5kv
Analyst:	YU	Nebulizer Gas Flow:	1.5L/min	Detector:	1.5kv
Sample:	PS-23 AR-36	CDL:	-20.0v	T. Flow:	0.2ml/min
M.W.:	4323.15	CDL Temp.:	250 °C	B. Conc.:	50%H2O/50%ACN
Lot. No.:	P230406-YW1070169	Block Temp.:	200 °C		

Biomatik
Tel: (519) 489-7195, (800) 836-8089
Fax: (519) 231-0140, (877) 221-3515
Email: info@biomatik.com
http://www.biomatik.com

HPLC REPORT

Sample Description:
Structure : PS-23 AR-36
Number : 0200046
Analyst : HCM
Lot No : P230506-YW1070169
Column : 4.6mm*250mm, SinoChrom ODS-BP
Solvent A : 0.1% trifluoroacetic in 100% acetonitrile
Solvent B : 0.1% trifluoroacetic in 100% water

Gradient :	A	B
0.01min	23%	77%
25min	48%	52%
25.1min	100%	0%
30.0min	STOP	

Flow rate : 1.0ml/min
Wavelength : 220nm
Volume : 5ul

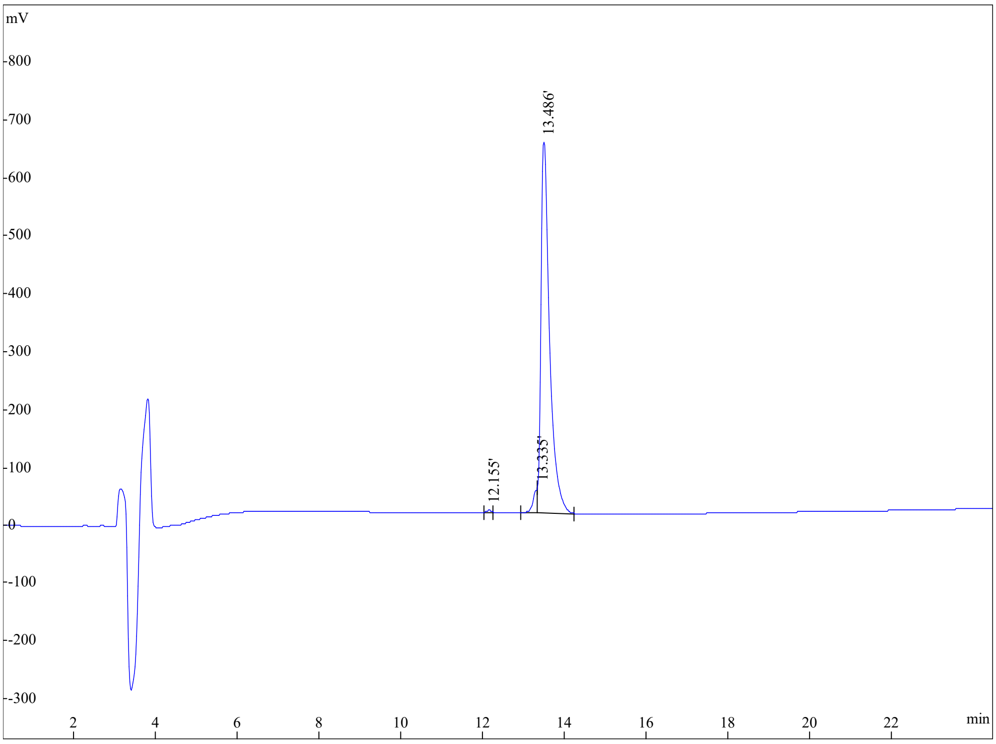

Rank	Time	Conc.	Area	Height
1	12.155	0.1983	17872	3026
2	13.335	3.274	295131	42578
3	13.486	96.53	8701834	638424
Total		100	9014837	684028

Biomatik
Tel: (519) 489-7195, (800) 836-8089
Fax: (519) 231-0140, (877) 221-3515
Email: info@biomatik.com
http://www.biomatik.com

Certificate of Analysis

**Date:**	2023-05-24
**Order Number:**	#SP230487
**Product Type:**	Chemically synthesized peptide
**Catalog Number:**	1070168
**Peptide Name:**	TA2-M1
**Sequence (N to C):**	5-FAM-YSSCQPQGFNCKVRPYGLPTIPCCRGLTRLSYSPGSTYGRCQRH
**MW:**	5297.91
**Salt Form:**	Trifluoroacetate (TFA Salt)
**Quantity:**	5.2mg
**Suggested Solvent:**	1.0mg peptide soluble in 1.0ml H2O
**Lot Number:**	P230406-YW1070168
**Appearance:**	Yellow lyophilized powder.
**Storage:**	Store lyophilized peptide at -20°C upon receipt. Reconstitute only the amount of peptide needed for immediate use.
**Limited Usage:**	For Research Use Only. Not for use in diagnostic procedures, or for administration to humans or animals.

**ASSAY**	**SPECIFICATION**	**ACTUAL**
**MW by MS**	5296.90	Conforms
**Purity by HPLC**	>95%	95.25%
**Peptide Content**	N/A	N/A
**TFA Content**	N/A	N/A
**Moisture Content**	N/A	N/A

Biomatik
Tel: (519) 489-7195, (800) 836-8089
Fax: (519) 231-0140, (877) 221-3515
Email: info@biomatik.com
http://www.biomatik.com

MS REPORT

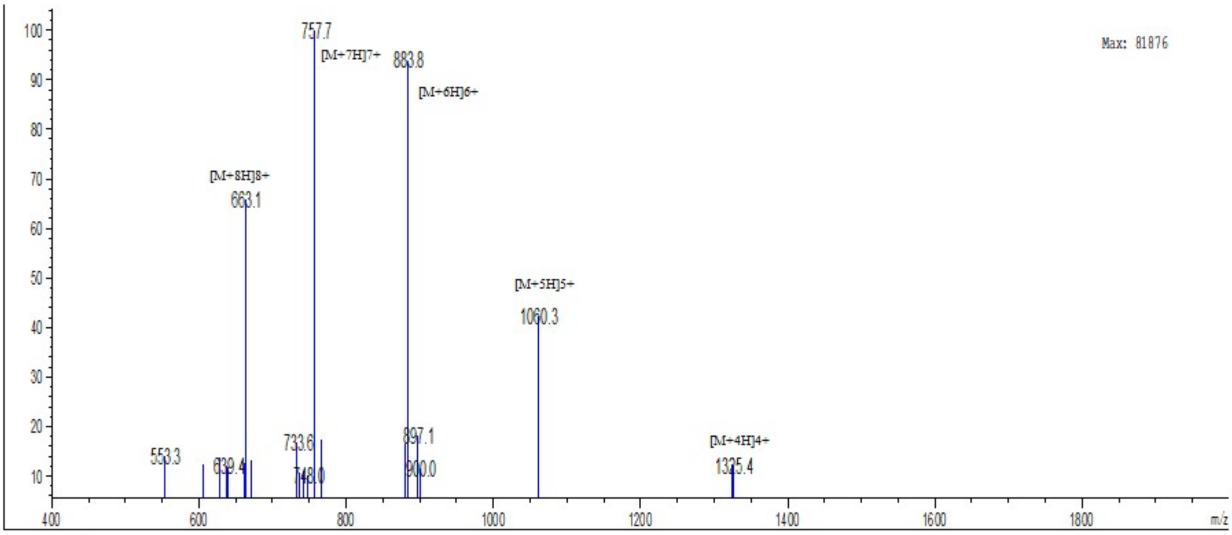

Sample Description		Instrument	Agilent-6125B		
Analyzed date:	2023-05-12	Probe:	ESI	Probe Bias:	+ 4.5kv
Analyst:	YU	Nebulizer Gas Flow:	1.5L/min	Detector:	1.5kv
Sample:	TA2-M1 YH-44	CDL:	-20.0v	T. Flow:	0.2ml/min
M.W.:	5297.91	CDL Temp.:	250 °C	B. Conc.:	50%H2O/50%ACN
Lot. No.:	P230406-YW1070168	Block Temp.:	200 °C		

Biomatik
Tel: (519) 489-7195, (800) 836-8089
Fax: (519) 231-0140, (877) 221-3515
Email: info@biomatik.com
http://www.biomatik.com

HPLC REPORT

Structure : TA2-M1 YH-44
Number : 010250011
Lot No : P230406-YW1070168
Column : 4.6×250mm,Kromasil 100-5 C4
Solvent A : 0.1% trifluoroacetic in 100% acetonitrile
Solvent B : 0.1% trifluoroacetic in 100% water
Gradient :

	A	B
0.01min	25%	75%
25min	50%	50%
25.1min	100%	0%
30min	STOP	

Flow rate : 1.0 mL/min
Wavelength : 220nm
Volume : 5ul

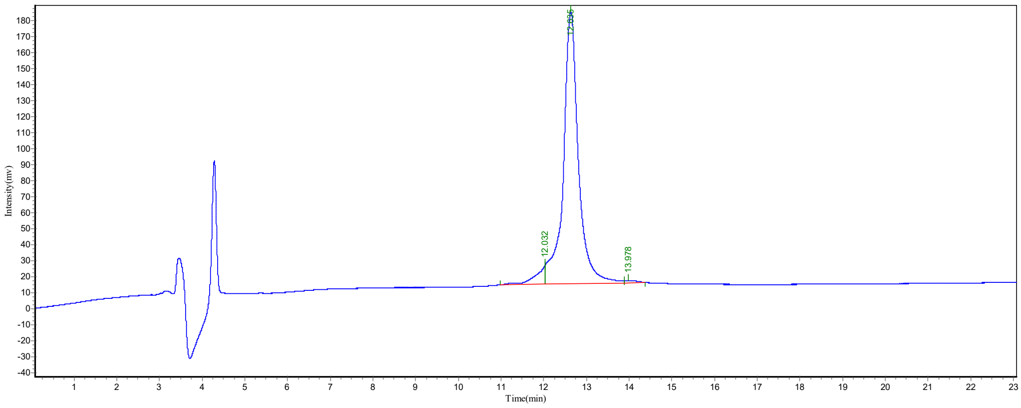

Peak No.	Ret Time	Height	Area	Conc.
1	12.032	11793.041	181961.500	4.1574
2	12.635	169892.625	4169121.750	95.2555
3	13.978	1360.043	25697.199	0.5871
Total				100.000

Integrated Life Science Products & Services
